# Effects of teachers’ interpersonal styles on psychological needs and engagement in college students in China

**DOI:** 10.3389/fpsyg.2026.1874740

**Published:** 2026-07-23

**Authors:** Rui Yuan, Yufei Qi, Jae-Hoon Hwang, Xinao Li, Yong-Gwan Song

**Affiliations:** 1 Department of Physical Education, University of Shanghai for Science and Technology, Shanghai, China; 2 Henan University of Technology, Zhengzhou, Henan, China; 3 Division of Smart Healthcare, Pukyong National University, Busan, Republic of Korea; 4 School of Physical Education, Yuxi Normal University, Yuxi, China

**Keywords:** autonomy support, control, engagement, interpersonal styles, psychological need

## Abstract

The present study examined the relationship between teachers’ autonomy support and control interpersonal styles, psychological needs, and engagement. Based on the online survey, participants were 734 college students in China. The results showed that teacher autonomy support was positively associated with psychological need satisfaction, whereas teacher control was positively associated with psychological need frustration. No significant relationship was found between teacher control and psychological need satisfaction. Psychological need satisfaction was positively associated with engagement (behavioral engagement, emotional engagement, cognitive engagement, and agentic engagement). Whereas Psychological need frustration was negatively associated with engagement (behavioral engagement, emotional engagement, and cognitive engagement). In the relationship between teacher interpersonal and engagement, perceived autonomy support was found to have mediating effect, while control was shown to have a partly mediating effect. Therefore, teachers interpersonal can improve students’ engagement (i.e., enthusiasm for class participation through interest, consideration, care, support), and effective communication to satisfy students’ psychological needs, which is important for teachers.

## Introduction

Teachers play a critical role in shaping students’ motivation, learning experiences, and engagement in educational settings. Within the framework of Self-Determination Theory (SDT; Ryan and Deci, 2000), teachers’ interpersonal styles are generally categorized as autonomy-supportive or controlling. Autonomy-supportive teaching involves acknowledging students’ perspectives and providing opportunities for choice and self-direction, whereas controlling teaching relies on pressure, external demands, and coercive strategies. These contrasting interpersonal styles are important because they influence how students experience the learning environment and engage in academic activities.

Autonomy-supportive teaching is characterized by behaviors that encourage students’ initiative, provide meaningful rationales, and support their interests and preferences (Michou et al., 2023; Reeve and Cheon, 2021; Cheon et al., 2012). Previous studies have consistently shown that autonomy-supportive teaching is associated with greater intrinsic motivation, academic achievement, persistence, and psychological well-being (Aelterman et al., 2016; Chen et al., 2019). When students perceive their teachers as supportive of their autonomy, they are more likely to invest effort in learning and participate actively in classroom activities.

In contrast, controlling teaching is characterized by pressuring language, excessive monitoring, and the use of rewards or punishments to regulate behavior (Reeve, 2002; Reeve and Cheon, 2021; De Meyer et al., 2016; Patall et al., 2013). Such practices may undermine students’ sense of personal agency and lead to psychological need frustration (Cheon et al., 2016; Haerens et al., 2015). Previous research has shown that controlling teaching is associated with negative educational outcomes, including emotional distress, lower motivation, reduced effort, and disengagement from learning activities (McDavid et al., 2014; Curran and Standage, 2017; Reeve and Cheon, 2021).

According to SDT, the satisfaction of three basic psychological needs—autonomy, competence, and relatedness—is essential for optimal functioning and well-being (Ryan and Deci, 2017). Psychological need satisfaction promotes adaptive outcomes such as motivation, engagement, and personal growth, whereas psychological need thwarting is associated with passivity, defensiveness, and maladjustment (Vansteenkiste and Ryan, 2013). Teachers’ interpersonal styles are considered important contextual factors that may either support or frustrate these psychological needs.

Learning engagement refers to students’ active involvement in learning activities and is commonly conceptualized as a multidimensional construct consisting of behavioral, emotional, cognitive, and agentic engagement. Engaged students demonstrate greater effort, enthusiasm, strategic learning, and proactive participation in educational activities. Previous research has shown that both teacher support and psychological need satisfaction are positively associated with learning engagement, whereas controlling educational environments are associated with lower levels of engagement (Jang et al., 2010, 2016; Reeve et al., 2020a,b; Matos et al., 2018).

Self-Determination Theory proposes that the influence of social environments on educational outcomes operates through the satisfaction or frustration of psychological needs. Consistent with this proposition, previous studies have reported that autonomy-supportive teaching is positively associated with psychological need satisfaction, which in turn is associated with higher levels of engagement and self-determined motivation (Michou et al., 2023; Patall et al., 2022; Reeve and Shin, 2020; Reeve et al., 2020a,b). These findings suggest that psychological needs may serve as an important mechanism linking teachers’ interpersonal styles to students’ engagement.

Although substantial evidence supports the relationships among autonomy-supportive teaching, psychological needs, and engagement, most previous studies have focused on primary and secondary school settings. Comparatively less attention has been given to higher education contexts, despite the fact that college students experience unique developmental challenges related to autonomy, identity formation, and career preparation. Consequently, further research is needed to examine whether the theoretical assumptions of SDT can be generalized to university students and to better understand how teachers’ interpersonal styles are associated with students’ engagement in higher education.

Therefore, the present study examined the associations among teachers’ autonomy-supportive and controlling interpersonal styles, psychological need satisfaction and thwarting, and learning engagement among Chinese college students. In addition, the mediating roles of psychological need satisfaction and thwarting were investigated within the framework of Self-Determination Theory. The findings are expected to contribute to a better understanding of the psychological processes underlying student engagement and to provide practical implications for promoting effective teaching practices in higher education.

## Materials and method

### Participants

The study participants were 734 students enrolled in universities in Zhengzhou Province, China, were selected as research participants using convenience sampling methods. The survey was conducted online, and participants were provided with an online survey link to easily respond to the survey anytime, anywhere. At the start of the online survey, it was explained that the collected data would not be used for any purposes other than research, and after ensuring strict confidentiality, participants who expressed interest in participating in the study were asked to respond to the questionnaire using a self-administration method. Frequency analysis was conducted to identify the demographic characteristics of the research participants, as shown in Table 1. The results revealed that out of the total participants, 434 (59.1%) were male students and 300 (40.9%) were female students. Regarding the grade level of the participants, there were 316 (43.1%) first-year students, 270 (36.8%) second-year students, and 105 (14.3%) third-year students, with 43 (5.9%) participants who did not respond. The gender of the instructors conducting the classes was identified as 519 (70.7%) male instructors and 215 (29.3%) female instructors.

**TABLE 1:** Characteristics of participants (*N* = 734).

Variable		Frequency (*n*)	Percentage (%)
Gender	Male	434	59.1
	Female	300	40.9
Grader	First	316	43.1
	Second	270	36.8
	Third	105	14.3
	Fourth	43	5.9
Total		734	100

The research was approved by the Institutional Review Board of University. Informed consent was obtained from each subject before participation in the study.

### Measures

All questionnaires were in the Chinese language, utilizing a previously validated Chinese-translated version of each English-language questionnaire Prior to the data collection, we professionally translated and back translated the grit scale, using Brislin’s (1980) step-by-step guidelines. Each measure used the same 7-point bipolar response scale (1 = strongly disagree, 7 = strongly agree).

### Teachers’ interpersonal styles

To measure students’ perceptions of teachers’ instructional styles, autonomy support and controlling style items were used. Autonomy support items were derived from the Learning Climate Questionnaire developed by Williams and Deci (1996), consisting of 6 items (e.g., “I feel that our professor understands me well”). Control items were adapted from the Control Style Questionnaire developed by Jang et al. (2009), comprising 4 items (e.g., “Our professor tries to control both my thoughts and actions”). Responses to the instructional strategy scale were rated on a 7-point scale (1 = Not at all true to 7 = Very true).

The validity of the instructional strategy scale was confirmed through confirmatory factor analysis. Items with standardized factor loadings below 0.50 were sequentially removed following theoretical review and psychometric evaluation. Consistent with previous recommendations (Hair et al., 2019; Kline, 2016), items with standardized factor loadings below 0.50 were considered for removal because they contributed weakly to their intended latent constructs and adversely affected convergent validity. After assessing the validity of the 2-factor, 10-item instructional strategy scale, one control item with low standardized loading was removed, resulting in 9 items with meaningful factor loadings. All standardized loadings were above 0.6. Generally, items with standardized loadings above 0.5 are considered significant. With all 9 items in the instructional strategy scale having standardized loadings above 0.6, they were all interpreted as meaningful.

Average Variance Extracted (AVE) and Composite Reliability (C.R.) values were calculated according to the given formulas. The AVE for autonomy support was 0.712, with a C.R. of 0.937, and for controlling style, the AVE was 0.614, with a C.R. of 0.816. AVE values above 0.5 indicate meaningful variance, and C.R. values above 0.7 indicate high internal consistency of the observed variables (Fornell and Larcker, 1981; Hair et al., 1998).

Upon assessing the fit of the 2-factor, 9-item instructional strategy scale, it was found that the TLI index (0.863) and RMSEA index (0.113) did not meet the standard criteria. However, the CFI index (0.901) and IFI index (0.901) were deemed satisfactory. To improve model fit, confirmatory factor analyses were re-examined. Based on theoretical justification and modification indices (M.I.), error covariances between conceptually related items within the same construct were permitted. The revised measurement models demonstrated acceptable fit according to recommended criteria (TLI = 0.901, RMSEA = 0.099, CFI = 928, IFI = 0.928). The reliability of the instructional strategy scale was verified, with α = 0.975 for autonomy support and α = 0.944 for control, demonstrating high levels of reliability.

### Psychological need

To measure the psychological needs perceived by college students, the Psychological Need Satisfaction Exercise Scale developed by Wilson et al. (2006) was employed. This scale distinguishes between autonomy, relatedness, and competence factors. Autonomy was measured using 5 items developed by Standage et al. (2006) (e.g., “I can choose activities in class that I enjoy”). Relatedness was assessed using 4 items developed by Richer and Vallerand (1998) (e.g., “My professors and classmates value me”). Competence was evaluated with 4 items developed by McAuley et al. (1989) (e.g., “I believe I am skilled at technical tasks”), totaling 14 items. Participants rated their psychological need satisfaction on a 7-point scale (1 = Not at all true to 7 = Very true).

Confirmatory factor analysis was conducted to assess the validity of the Psychological Need Satisfaction Scale. Items with standardized factor loadings below 0.50 were sequentially removed following theoretical review and psychometric evaluation. Consistent with previous recommendations (Hair et al., 2019; Kline, 2016), items with standardized factor loadings below 0.50 were considered for removal because they contributed weakly to their intended latent constructs and adversely affected convergent validity. After examining the validity of the 3-factor, 13-item scale, one relatedness item with low standardized loading was removed, resulting in 12 items across 3 factors with meaningful factor loadings. All standardized loadings were above 0.6. Upon calculating the Average Variance Extracted (AVE) and Composite Reliability (C.R.) values, the AVE for autonomy was 0.608, with a C.R. of 0.855. For competence, the AVE was 0.548, with a C.R. of 0.823, and for relatedness, the AVE was 0.451, with a C.R. of 0.700. To improve convergent validity and construct reliability, items with relatively low standardized factor loadings (i.e., relatedness item) were removed following confirmatory factor analysis. The measurement model was re-estimated after each modification. The final relatedness model demonstrated acceptable factor loadings, AVE, and C.R., exceeding the recommended thresholds for convergent validity and construct reliability (relatedness AVE = 0.512, C.R. = 774).

Assessment of the fit of the 3-factor, 12-item Psychological Need Satisfaction Scale revealed that the TLI index (0.877) and RMSEA index (0.153) did not meet the standard criteria. However, the CFI index (0.905), IFI index (0.905), and NFI index (0.900) were satisfactory. Based on theoretical justification and modification indices (M.I.), error covariances between conceptually related items within the same construct were permitted. The revised measurement models demonstrated acceptable fit according to recommended criteria (TLI = 0.904, RMSEA = 0.100, CFI = 931, IFI = 0.931, NFI = 0.907). Reliability analysis of psychological need satisfaction showed high levels of reliability, with α = 0.939 for autonomy, α = 0.953 for competence, and α = 0.919 for relatedness.

### Psychological need thwarting

To assess college students perceived psychological need thwarting, the Psychological Need Thwarting Scale developed by Bartholomew et al. (2011) was utilized. This scale comprises autonomy need thwarting with 4 items (e.g., “I feel like I participate in class because of coercion or pressure”), competence need thwarting with 4 items (e.g., “In class, I often encounter situations where I feel incompetent”), and relatedness need thwarting with 4 items (e.g., “I feel rejected when I’m with friends”), totaling 12 items across 3 factors. Participants rated their psychological need thwarting on a 7-point scale (1 = Not at all true to 7 = Very true).

Confirmatory factor analysis was conducted to assess the validity of the Psychological Need Thwarting Scale. After examining the validity of the 3-factor, 12-item scale, one autonomy thwarting item with low standardized loading was removed, resulting in 11 items across 3 factors with meaningful factor loadings. All standardized loadings for the 12 items of psychological need thwarting were above 0.6, indicating that all items were meaningful.

Upon calculating the Average Variance Extracted (AVE) and Composite Reliability (C.R.) values, the AVE for autonomy thwarting was 0.550, with a C.R. of 0.785. For relatedness thwarting, the AVE was 0.799, with a C.R. of 0.914, and for competence thwarting, the AVE was 0.594, with a C.R. of 0.854.

Assessment of the fit of the 3-factor, 11-item Psychological Need Thwarting Scale revealed that the RMSEA index (0.116) did not meet the standard criteria. However, the CFI index (0.961), TLI index (0.948), IFI index (0.961), and NFI index (0.958) were satisfactory. Based on theoretical justification and modification indices (M.I.), error covariances between conceptually related items within the same construct were permitted. The revised measurement models demonstrated acceptable fit according to recommended criteria (TLI = 0.956, RMSEA = 0.099, CFI = 970, IFI = 0.970, NFI = 0.964). Reliability analysis of the Psychological Need Thwarting Scale showed high levels of reliability, with α = 0.920 for autonomy thwarting, α = 0.977 for relatedness thwarting, and α = 0.951 for competence thwarting.

### Learning engagement

To measure college students’ perceived learning engagement, the Learning Engagement Scale questionnaire developed by Reeve and Tseng (2011) was utilized. This scale consists of behavior engagement with 5 items (e.g., “I actively participate in class”), emotional engagement with 5 items (e.g., “I find the class interesting and enjoyable”), cognitive engagement with 4 items (e.g., “I try to understand the material learned in class”), and agentic engagement with 5 items (e.g., “I suggest ideas or opinions to the teacher for a better class”), totaling 19 items across 4 factors. Participants rated their learning engagement on a 7-point scale (1 = Not at all true to 7 = Very true).

Confirmatory factor analysis was conducted to assess the validity of the Classroom Engagement Scale. Items with standardized factor loadings below 0.50 were sequentially removed following theoretical review and psychometric evaluation. Consistent with previous recommendations (Hair et al., 2019; Kline, 2016), items with standardized factor loadings below 0.50 were considered for removal because they contributed weakly to their intended latent constructs and adversely affected convergent validity. After examining the validity of the 4-factor, 19-item scale, one emotional engagement item with low standardized loading was removed, resulting in 18 items across 4 factors with meaningful factor loadings.

Upon calculating the Average Variance Extracted (AVE) and Composite Reliability (C.R.) values, the AVE for behavior engagement was 0.722, with a C.R. of 0.928. For emotional engagement, the AVE was 0.794, with a C.R. of 0.939. For cognitive engagement, the AVE was 0.342, with a C.R. of 0.644, and for agentic engagement, the AVE was 0.586, with a C.R. of 0.875.

To improve convergent validity and construct reliability, items with relatively low standardized factor loadings (i.e., cognitive item) were removed following confirmatory factor analysis. The measurement model was re-estimated after each modification. The final relatedness model demonstrated acceptable factor loadings, AVE, and C.R., exceeding the recommended thresholds for convergent validity and construct reliability (relatedness AVE = 0.508, C.R. = 776).

Assessment of the fit of the 4-factor, 18-item learning engagement scale revealed that the RMSEA index (0.123) did not meet the standard criteria. However, the CFI index (0.924), TLI index (0.910), NFI index (0.928), and IFI index (0.924) were satisfactory. Based on theoretical justification and modification indices (M.I.), error covariances between conceptually related items within the same construct were permitted. The revised measurement models demonstrated acceptable fit according to recommended criteria (TLI = 0.920, RMSEA = 0.097, CFI = 935, IFI = 0.935, NFI = 0.937). Reliability analysis of the learning engagement scale showed high levels of reliability, with α = 0.955 for behavior engagement, α = 0.961 for emotional engagement, α = 0.940 for cognitive engagement, and α = 0.960 for agentic engagement.”

### Data analysis

The data were analyzed using SPSS and AMOS statistical software. Frequency and descriptive statistics analyses were conducted in SPSS to understand the characteristics of the participants and the characteristics of each item. Additionally, reliability analysis was performed to assess the reliability of the survey instruments, and correlation analysis was used to examine the relationships between variables. AMOS software was utilized for validity analysis (confirmatory factor analysis) and path analysis. Through path analysis, the influences of autonomy support and control on students’ psychological needs and learning engagement was examined. Bootstrap analysis was employed as an additional step to verify the mediating effects (Baron and Kenny, 1986). All statistical significance levels were set at 0.05.

## Results

### Descriptive statistics and correlation analysis

The descriptive statistics of the study variables revealed the following: Autonomy support was found to be 6.03 (SD = 1.25), control was 3.35 (SD = 1.44), autonomy was 5.55 (SD = 1.28), competence was 5.43 (SD = 1.22), relatedness was 4.75 (SD = 1.15), autonomy frustration was 3.48 (SD = 1.45), relatedness frustration was 2.42 (SD = 1.57), competence frustration was 3.03 (SD = 1.60), behavioral engagement was 5.61 (SD = 1.17), emotional engagement was 5.29 (SD = 1.04), cognitive engagement was 5.29 (SD = 1.14), and agentic engagement was 5.28 (SD = 1.30).

To investigate the relationships between teachers’ interpersonal styles, psychological need satisfaction, thwarting, and learning engagement variables, correlation analysis was conducted. As presented in Table 2, autonomy support showed significant positive correlations with autonomy (*r* = 0.525, *p* < 0.01), competence (*r* = 0.488, *p* < 0.01), relatedness (*r* = 0.325, *p* < 0.01). In contrast, autonomy support exhibited significant negative correlations with autonomy thwarting (*r* = −0.187, *p* < 0.01), relatedness thwarting (*r* = −0.243, *p* < 0.01), competence thwarting (*r* = −0.168, *p* < 0.01). Moreover, autonomy support demonstrated significant positive correlations with behavioral engagement (*r* = 0.499, *p* < 0.01), emotional engagement (*r* = 0.457, *p* < 0.01), cognitive engagement (*r* = 0.402, *p* < 0.01), agentic engagement (*r* = 0.420, *p* < 0.01).

**TABLE 2:** Intercorrelations and descriptive statistics for the variables.

Variable	1	2	3	4	5	6	7	8	9	10	11	12
1. Autonomy support	1	−0.034	0.525**	0.488**	0.325**	−0.187**	−0.243**	−0.168**	0.499**	0.457**	0.402**	0.420**
2. Control		1	−0.092*	−0.088*	0.048	0.411**	0.379**	0.295**	−0.088*	−0.023	−0.004	−0.006
3. Autonomy			1	0.785**	0.509**	−0.123**	−0.265**	−0.246**	0.650**	0.607**	0.578**	0.576**
4. Competence				1	0.629**	−0.103**	−0.183**	−0.199**	0.680**	0.659**	0.606**	0.621**
5. Relatedness					1	0.116**	0.062	0.000	0.506**	0.541**	0.504**	0.518**
6. Autonomy frustration						1	0.674**	0.582**	−0.121**	−0.005	−0.007	−0.047
7. Relatedness frustration							1	0.730**	−0.265**	−0.139**	−0.130**	−0.145**
8. Competence frustration								1	−0.213**	−0.112**	−0.118**	−0.209**
9.Behavior Engagement									1	0.909**	0.860**	0.815**
10. Emotion engagement										1	0.882**	0.815**
11. Conitive engagement											1	0.775**
12. Agentic engagement												1
Mean	6.03	3.35	5.55	5.43	4.75	3.48	2.42	3.03	5.61	5.29	5.29	5.28
Standard deviation	1.25	1.44	1.28	1.22	1.15	1.45	1.57	1.60	1.17	1.04	1.14	1.30

Any correlation *p < 0.05,

**p < 0.01.

Control was negatively correlated with autonomy (*r* = −0.092, *p* < 0.05) and competence (*r* = −0.088, *p* < 0.05), and positively correlated with autonomy thwarting (*r* = 0.411, *p* < 0.01), relatedness thwarting (*r* = 0.379, *p* < 0.01), competence thwarting (*r* = 0.295, *p* < 0.01). Additionally, control was negatively correlated with behavioral engagement (*r* = −0.088, *p* < 0.05).

Autonomy showed significant negative correlations with autonomy thwarting (*r* = −0.123, *p* < 0.01), relatedness thwarting (*r* = −0.265, *p* < 0.01), competence thwarting (*r* = −0.246, *p* < 0.01). And autonomy significant positive correlations with behavioral engagement (*r* = 0.650, *p* < 0.01), emotional engagement (*r* = 0.607, *p* < 0.01), cognitive engagement (*r* = 0.578, *p* < 0.01), agentic engagement (*r* = 0.576, *p* < 0.01).

Competence also showed significant negative correlations with autonomy thwarting (*r* = −0.103, *p* < 0.01), relatedness thwarting (*r* = −0.183, *p* < 0.01), competence thwarting (*r* = −0.199, *p* < 0.01). And competence significant positive correlations with behavioral engagement (*r* = 0.680, *p* < 0.01), emotional engagement (*r* = 0.659, *p* < 0.01), cognitive engagement (*r* = 0.606, *p* < 0.01), agentic engagement (*r* = 0.621, *p* < 0.01).

Relatedness was negatively correlated with autonomy thwarting (*r* = 0.116, *p* < 0.01) and positively correlated with behavioral engagement (*r* = 0.506, *p* < 0.01), emotional engagement (*r* = 0.541, *p* < 0.01), cognitive engagement (*r* = 0.504, *p* < 0.01), agentic engagement (*r* = 0.518, *p* < 0.01).

Autonomy thwarting was negatively correlated with behavioral engagement (*r* = −0.121, *p* < 0.05). Relatedness thwarting showed significant negative correlations with behavioral engagement (*r* = −0.265, *p* < 0.01), emotional engagement (*r* = −0.139, *p* < 0.01), cognitive engagement (*r* = −0.130, *p* < 0.01), agentic engagement (*r* = −0.145, *p* < 0.01).

Competence thwarting demonstrated significant negative correlations with behavioral engagement (*r* = −0.213, *p* < 0.01), emotional engagement (*r* = −0.112, *p* < 0.01), cognitive engagement (*r* = −0.118, *p* < 0.01), agentic engagement (*r* = −0.209, *p* < 0.01).

### Path analysis

To verify the influence of teachers’ interpersonal styles on psychological need satisfaction, thwarting, and learning engagement, path analysis was conducted.

The path analysis results (see Table 3) revealed that autonomy support was significantly and positively associated with psychological need satisfaction (autonomy support → psychological need satisfaction β = 0.445, *p* < 0.001) and a significant negative direct effect on psychological need thwarting (autonomy support → psychological need thwarting β = −0.231, *p* < 0.001). Conversely, control was significantly and positively associated with psychological need thwarting (control → psychological need thwarting β = 0.377, *p* < 0.001) and a non-significant direct effect on psychological need satisfaction (control → psychological need satisfaction β = −0.030, ns).

**TABLE 3:** Path analysis of effect of dependent variable on independent.

Independent	Path	Dependent variable	Estimate	S.E.	C.R.	SRW	*P*
Autonomy support	→	Needs satisfaction	0.445	0.027	16.474	0.519	***
Control	→	Needs Frustration	0.377	0.031	12.233	0.401	***
Autonomy support	→	Needs frustration	−0.231	0.036	−6.479	−0.213	***
Control	→	Needs satisfaction	−0.030	0.023	−1.271	−0.040	0.204
Needs satisfaction	→	Behavioral engagement	0.753	0.029	26.380	0.688	***
Needs satisfaction	→	Emotional engagement	0.677	0.026	25.614	0.691	***
Needs satisfaction	→	Cognitive engagement	0.692	0.030	22.715	0.646	***
Needs satisfaction	→	Agentic engagement	0.788	0.034	22.974	0.646	0.501
Needs frustration	→	Behavioral engagement	−0.106	0.022	−4.715	−0.123	***
Needs frustration	→	Emotional engagement	0.006	0.021	0.305	0.008	0.760
Needs frustration	→	Cognitive engagement	0.001	0.024	0.058	0.002	0.954
Needs frustration	→	Agentic engagement	−0.052	0.027	−1.921	−0.054	0.055

***p < 0.001.

Psychological need satisfaction was positively associated with behavioral engagement (psychological need satisfaction → behavioral engagement β = 0.753, *p* < 0.001), emotional engagement (psychological need satisfaction → emotional engagement β = 0.677, *p* < 0.001), and cognitive engagement (psychological need satisfaction → cognitive engagement β = 0.692, *p* < 0.001). Psychological need thwarting was negatively associated with behavioral engagement (psychological need thwarting → behavioral engagement β = −0.105, *p* < 0.001).

To assess the fit of the analyzed path model, fitness indices were examined. As shown in results, the Root Mean Square Error of Approximation (RMSEA) was below the threshold of 0.1 (RMSEA = 0.094), indicating an acceptable fit. The Comparative Fit Index (CFI), Tucker-Lewis Index (TLI), Incremental Fit Index (IFI), and Normed Fit Index (NFI) were all above 0.9, demonstrating acceptable model fit. Relative Fit Index (RFI) evaluates the model’s fit compared to the independence model and indicates how well the model explains the data compared to the null model. On the other hand, Absolute Fit Index (AFI) evaluates the fit of the theoretical model absolutely, without comparing it to the independence model. From these perspectives, the fit of the research model appears to be satisfactory.

The direct, indirect, and total effects of teachers’ instructional styles on psychological need satisfaction, thwarting, and learning engagement variables are presented in Table 4. The results indicated that the total effect of autonomy support on psychological need thwarting was −0.213 (direct effect: −0.213), and on psychological need satisfaction was 0.519 (direct effect: 0.519). The total effect of control on psychological need thwarting was 0.401 (direct effect: 0.401), and on psychological need satisfaction was −0.040 (direct effect: −0.040). Moreover, autonomy support was indirectly associated with agentic engagement (β = 0.347, 95% CI [0.299, 0.429]), cognitive engagement (β = 0.335, 95% CI [0.243, 0.366]), emotional engagement (β = 0.357, 95% CI [0.239, 0.360]), and behavioral engagement (β = 0.383, 95% CI [0.300, 0.421]) through psychological need satisfaction. Control was indirectly associated with agentic engagement (β = −0.048, 95% CI [−0.085, −0.004]), cognitive engagement (β = −0.025, 95% CI [−0.057, 0.016]), emotional engagement (β = −0.024, 95% CI [−0.053, 0.017]), and behavioral engagement (β = −0.077, 95% CI [−0.100, −0.026]) through psychological need satisfaction.

**TABLE 4:** Total, indirect and direct effect of hypothesis model.

Path	Direct β	Indirect β	Total β	95% bootstrap CI	Significance
Autonomy support→ needs frustration	−0.213		−0.213		*p* < 0.001
Autonomy support→ needs satisfaction	0.519		0.519		*p* < 0.001
Control→ needs frustration	0.401		0.401		*p* < 0.001
Control→ needs satisfaction	−0.040		−0.040		ns
Autonomy support→ agentic engagement	0.000	0.347	0.347	[0.299, 0.429]	*p* < 0.05
Autonomy support→ cognitive engagement	0.000	0.335	0.335	[0.243, 0.366]	*p* < 0.05
Autonomy support→ emotional engagement	0.000	0.357	0.357	[0.239, 0.360]	*p* < 0.05
Autonomy support→ behavioral engagement	0.000	0.383	0.383	[0.300, 0.421]	*p* < 0.05
Control→ agentic engagement	0.000	−0.048	−0.048	[−0.085, −0.004]	*p* < 0.05
Control→ cognitive engagement	0.00.	−0.025	−0.025	[−0.057,0.016]	Ns
Control→emotional engagement	0.000	−0.024	−0.024	[−0.053, 0.017]	Ns
Control→ behavioral engagement	0.000	−0.077	−0.077	[−0.100, −0.026]	*p* < 0.05
Needs frustration→ agentic engagement	−0.054		−0.054		Ns
Needs frustration→ cognitive engagement	0.002		0.002		Ns
Needs frustration→ emotional engagement	0.008		0.008		Ns
Needs frustration→ behavioral engagement	−0.123		−0.123		*p* < 0.001
Needs satisfaction→ agentic engagement	0.646		0.646		*p* < 0.001
Needs satisfaction→ Cognitive engagement	0.646		0.646		*p* < 0.001
Needs satisfaction→ emotional engagement	0.691		0.691		*p* < 0.001
Needs satisfaction→ behavioral engagement	0.688		0.688		*p* < 0.001

Additionally, psychological need thwarting and learning engagement showed total effects of −0.054 (agentic engagement), 0.002 (cognitive engagement), 0.008 (emotional engagement), and −0.123 (behavioral engagement) without indirect effects. Similarly, psychological need satisfaction and learning engagement showed total effects of 0.646 (agentic engagement), 0.646 (cognitive engagement), 0.691 (emotional engagement), and 0.688 (behavioral engagement) without indirect effects.

Finally, bootstrapping confirmed the significance of the mediating effects of psychological need satisfaction and thwarting. Indirect associations were evaluated using bias-corrected bootstrapping (5,000 resamples). An indirect association was considered significant when the 95% bootstrap confidence interval did not include zero. The paths autonomy support → psychological need satisfaction → agentic engagement, autonomy support → psychological need satisfaction → cognitive engagement, autonomy support → psychological need satisfaction → emotional engagement, and autonomy support → psychological need satisfaction → behavioral engagement all exhibited significant indirect effects at the 95% confidence level with *p* < 001. Furthermore, the path control → psychological need thwarting → behavioral engagement also showed a significant indirect effect at the 95% confidence level with *p* < 0.05. These findings suggest that psychological need satisfaction may function as a mediating mechanism linking autonomy-supportive teaching and learning engagement.

## Discussion

The present study examined the associations among teachers’ interpersonal styles, psychological need satisfaction and frustration, and learning engagement among Chinese college students. In addition, the mediating roles of psychological needs in the relationships between teachers’ interpersonal styles and learning engagement were investigated. The findings indicated that autonomy-supportive teaching was positively associated with psychological need satisfaction and learning engagement, whereas controlling teaching was positively associated with psychological need frustration and showed relatively weak associations with learning engagement. Furthermore, psychological need satisfaction emerged as an important mechanism linking autonomy-supportive teaching to students’ learning engagement. These findings underscore the importance of fostering autonomy-supportive learning environments that facilitate students’ psychological need satisfaction and active engagement in learning.

According to Self-Determination Theory (Ryan and Deci, 2017), the satisfaction of the basic psychological needs for autonomy, competence, and relatedness is essential for optimal motivation, engagement, and well-being. The present findings are consistent with this theoretical perspective, demonstrating that autonomy-supportive teaching was positively associated with psychological need satisfaction, whereas controlling teaching was positively associated with psychological need frustration. Autonomy-supportive teaching involves acknowledging students’ perspectives, providing meaningful rationales, offering opportunities for choice, and encouraging self-initiation (Reeve, 2002; Reeve and Cheon, 2021). Such instructional practices create learning environments that support students’ psychological needs and facilitate adaptive motivational processes. In contrast, controlling teaching relies on external pressures, rewards, punishments, and excessive regulation, which may undermine students’ sense of autonomy and contribute to need frustration (Bartholomew et al., 2010; Cheon et al., 2019; Costa et al., 2015; Jang et al., 2009).

The present findings further indicated that psychological need satisfaction was positively associated with all dimensions of learning engagement. Students who experience greater autonomy, competence, and relatedness are more likely to invest effort in learning, experience positive emotions, engage in deeper cognitive processing, and actively contribute to classroom activities. These findings are consistent with previous studies demonstrating that psychological need satisfaction serves as an important motivational resource that promotes engagement and academic functioning (Patall et al., 2019; Reeve et al., 2020a,b, 2022). Conversely, psychological need frustration was negatively associated with behavioral engagement, suggesting that unmet psychological needs may contribute to disengagement and reduced participation in learning activities.

Learning engagement is a multidimensional construct encompassing behavioral, emotional, cognitive, and agentic components (Reeve, 2013). Students with higher levels of engagement tend to demonstrate greater interest, enthusiasm, persistence, strategic learning, and proactive participation in educational activities. Consistent with previous research, the present findings suggest that autonomy-supportive teaching is positively associated with students’ engagement through the satisfaction of their psychological needs (Benlahcene et al., 2022; Michou et al., 2023; Patall et al., 2022). These findings highlight the importance of supportive instructional practices that encourage students to take an active role in their learning process.

An important contribution of the present study is the finding that autonomy-supportive teaching and controlling teaching did not demonstrate equivalent associations with students’ motivational experiences. Although both interpersonal styles were related to psychological needs, autonomy support exhibited substantially stronger associations with psychological need satisfaction and learning engagement than did controlling teaching (Reeve et al., 2020a,b). In contrast, the associations involving controlling teaching were relatively small and primarily linked to psychological need frustration. Notably, several indirect associations involving controlling teaching were statistically significant but demonstrated relatively small effect sizes. These findings suggest that promoting autonomy-supportive teaching practices may be more important for enhancing student engagement than merely reducing controlling instructional behaviors.

From a theoretical perspective, the present findings extend previous SDT-based research by highlighting the asymmetric roles of autonomy support and control within higher education settings. Traditional SDT research often conceptualizes autonomy support and control as opposing interpersonal styles; however, the current findings suggest that autonomy support may play a more influential role in facilitating engagement than control does in undermining it. This finding contributes to the literature by extending the application of SDT to Chinese college students and by demonstrating that positive motivational resources may be particularly important for fostering engagement in higher education contexts.

The findings also have important practical implications. University instructors should create learning environments that support students’ autonomy by acknowledging their perspectives, providing meaningful rationales, offering opportunities for choice, and encouraging active participation. Such practices may help satisfy students’ psychological needs and promote higher levels of engagement. In contrast, excessive reliance on controlling instructional strategies may contribute to psychological need frustration and lower-quality learning experiences. Therefore, teacher education and professional development programs should emphasize autonomy-supportive instructional practices as a means of enhancing student engagement and academic development.

This study has several limitations. First, the cross-sectional design does not permit causal inferences regarding the relationships among the study variables. Future research should employ longitudinal or experimental designs to further examine the directionality of these associations. Second, all variables were measured using self-report questionnaires administered to the same participants at a single point in time. Consequently, common method variance may have influenced the observed associations among the constructs. Future studies should incorporate multi-source data collection and additional procedural or statistical controls to minimize potential common method bias.

Third, although the revised measurement models demonstrated acceptable psychometric properties, several issues related to measurement validity should be considered. The instruments were originally developed and validated in different cultural and educational contexts and were subsequently translated and adapted for use with Chinese college students. Therefore, cultural differences, language interpretation, and contextual characteristics may have influenced the measurement performance of some constructs. In addition, several items with relatively low factor loadings were removed during the validation process to improve model fit and construct reliability. Although these modifications improved the psychometric quality of the measures, they may have reduced the breadth of content represented by certain constructs. Future research should continue to evaluate the cross-cultural validity, measurement invariance, and construct coverage of these instruments across diverse educational settings.

In conclusion, the present study provides evidence that autonomy-supportive teaching is positively associated with psychological need satisfaction and learning engagement among Chinese college students. Psychological need satisfaction emerged as an important mechanism linking teachers’ interpersonal styles and students’ engagement. The findings underscore the value of autonomy-supportive teaching practices and contribute to a better understanding of the motivational processes underlying learning engagement in higher education.

## Conclusion

This study examined how university students perceive teachers’ interpersonal styles and how these perceptions are associated with psychological needs and learning engagement. The findings suggest that the level and quality of learning engagement are closely related to students’ psychological need satisfaction. In particular, autonomy-supportive teaching was positively associated with psychological need satisfaction and learning engagement, whereas controlling teaching was associated with psychological need frustration and lower-quality engagement.

Based on these findings, several directions for future research are suggested. First, future studies should examine how diverse teaching strategies, beyond autonomy support and control, are associated with psychological need satisfaction, need frustration, and learning engagement. Future research should also investigate how psychological need experiences are related to broader student outcomes, such as autonomous motivation, emotion, achievement, creativity, and social development. Second, future studies should identify which specific teaching strategies are most strongly associated with autonomy satisfaction and high-quality engagement. Finally, although cross-sectional studies remain useful for examining associations among variables, longitudinal and ecological approaches are needed to clarify developmental processes and enhance ecological validity.

## Data Availability

The original contributions presented in this study are included in this article/supplementary material, further inquiries can be directed to the corresponding authors.
